# Oral Dyskinesia in a Pediatric Patient Following Concurrent Use of Neuroleptics and Stimulants: Treatment Strategy Considerations to Avert Avoidable Adverse Side Effects

**DOI:** 10.7759/cureus.38294

**Published:** 2023-04-29

**Authors:** Rangin Haji Rahman, Sanjaya Dharmapuri

**Affiliations:** 1 Medicine, Saint James School of Medicine, Park Ridge, USA; 2 Psychiatry and Behavioral Sciences, Garfield Park Behavioral Hospital, Chicago, USA

**Keywords:** second-generation antipsychotics, pseudo-tardive, withdrawal dyskinesia, withdrawal-emergent dyskinesia, antipsychotic management, oral dyskinesia

## Abstract

Withdrawal-emergent dyskinesia is a movement disorder that emerges following sudden discontinuation or rapid taper of antipsychotic medication. It is infrequently identified and typically resolves within a few weeks from symptom onset. This case report describes a unique case of reversible oral dyskinesia in a 13-year-old male in the context of concurrent neuroleptic withdrawal and stimulant titration. The extant literature describing tardive dyskinesia is well-established; however, few studies have thoroughly examined withdrawal-emergent dyskinesia and other tardive syndromes. This report highlights the importance of clinician awareness as far as the potential for extrapyramidal symptoms and withdrawal-emergent adverse effects in concomitant management of antipsychotics and stimulants in the child and adolescent populations and may help inform future treatment and management of disorders that would indicate the concurrent use of these psychotropics.

## Introduction

Tardive dyskinesia (TD) is a disabling and irreversible adverse effect of long-term exposure to dopamine receptor-blocking agents, namely, first and second-generation antipsychotic medications [1]. It is characterized by abnormal involuntary movements of the face, lips, tongue, and jaw [1]. Withdrawal-emergent dyskinesia (WED), also known as withdrawal extrapyramidal symptoms (EPS), is considered a subtype of TD and is characterized by abnormal movements of the trunk, extremities, face, and neck, following a rapid reduction in dose or sudden discontinuation of antipsychotic medications [2]. While TD has a low rate of remission, in WED, general improvement may be seen in a shorter period of one to two months [2]. Some studies also note the emergence of WED as a predictor of TD development with long-term antipsychotic medication use [3].

Childhood and adolescent psychiatry has seen an increase in neuroleptic use as select neuroleptics have been approved for the treatment of a wide range of disorders [4]. These include severe conduct problems and disruptive behavior disorders, psychotic disorders, bipolar disorder, Tourette’s syndrome, and severe irritability in children with autism spectrum disorders [4]. Antipsychotic agents present a considerable number of side effects, of which TD is the most severe in the setting of long-standing use. Spontaneous movements developing in the context of sudden discontinuation or rapid taper of antipsychotics raise the possibility of WED. In this report, we examine a case of oral dyskinesia in a 13-year-old male.

## Case presentation

A 13-year-old male with a psychiatric history of disruptive mood dysregulation disorder (DMDD) and attention-deficit hyperactivity disorder (ADHD) presented with increased aggression and homicidal ideation toward a family member and destruction of property. The patient’s medication history before admission included clonidine 0.1 mg by mouth (PO) every 12 hours; fluoxetine 40 mg PO daily; metformin 250 mg PO twice a day with meals for pre-diabetes; and ziprasidone 40 mg PO twice a day with meals (two-month duration preceding hospitalization). Methylphenidate 18 mg PO every morning was initiated during hospitalization. Past medication history included atomoxetine and quetiapine, both of which were initiated three months preceding hospitalization, for a duration of 30 days. The dose of methylphenidate varied throughout his stay, beginning at 18 mg on admission, and reaching a maximum titrated dose of 54 mg on his fourth day of hospitalization. The dose of clonidine was increased on the sixth day of hospitalization to 0.1 mg PO three times per day as an adjunctive treatment for ADHD symptoms and worsening inattention.

On the fourth day of his hospitalization, the patient demonstrated severe hyperactivity and impulsiveness on examination and continued to show low frustration tolerance. As such, ziprasidone was discontinued and a trial of sodium valproate 250 mg PO twice a day was initiated to achieve better mood stabilization. EPS in the form of oral dyskinesia was noted later that same day. On the fifth day, an increase in facial grimacing and mouth twitching was noted and he was given 1 mg of benztropine to address the possible atypical presentation of acute-onset EPS, as well as an as-needed dose of ziprasidone 20 mg PO as a bridge to mitigate the suspected withdrawal-emergent symptoms resulting from the rapid discontinuation. He still reported having elevated energy and anxiety. The dose of fluoxetine was titrated down to 20 mg and methylphenidate was reduced to 18 mg in an attempt to reduce the adverse effect of anxiety. On day nine, the patient’s EPS, as well as anxiety, were diminished; however, his symptoms of inattention worsened. Given concerns for overactivation, methylphenidate was tapered to discontinuation and a trial of bupropion (sustained release 37 mg PO everyday) was initiated to compensate for concerns for dopaminergic withdrawal and hypersensitivity to potential emergent withdrawal symptoms from discontinuation of the antipsychotic. The patient had subsequent remission of extrapyramidal and tardive symptoms.

## Discussion

The pathophysiology of TD is yet to be clearly elucidated and several mechanisms have been postulated in the literature. The most prevalent mechanism is postsynaptic dopamine receptor hypersensitivity as a consequence of long-term administration of antipsychotics [5]. Neuroleptics exert their effects predominantly on the dopamine (D2) receptor, and dopamine hypersensitivity results from prolonged blockade of this receptor which induces a compensatory upregulation of receptors over time [5]. The D2 receptors are located in the mesocortical and mesolimbic dopaminergic pathways, which are involved in reward and cognition. D2 receptors are also found in the tuberoinfundibular and nigrostriatal pathways, which are involved in the regulation of prolactin secretion and motor planning, respectively. Dopaminergic blockade in the nigrostriatal pathway produces the characteristic EPS associated with prolonged antipsychotic exposure. Atypical antipsychotics are associated with the lowest risk of EPS as they act predominantly on the mesolimbic dopamine pathway, as opposed to the nigrostriatal pathway [5]. This is likely due to their antagonism of the serotonin (5HT2a) receptor in addition to the D2 receptor [5]. Furthermore, atypical antipsychotics have a lower binding affinity to D2 receptors and rapidly disassociate, thus lowering the propensity for EPS [6]. Conversely, first-generation antipsychotics bind with greater affinity than dopamine to the D2 receptor, in turn producing a more potent EPS profile [6]. Although the risk and incidence of EPS decrease with atypical antipsychotics compared to typical antipsychotics, the risk persists.

Pseudo-tardive manifestations and withdrawal-emergent symptoms have not been well-characterized in the literature. Pseudo-tardive dyskinesia shares the same features as TD; however, it may be attributed to alternate etiologies. Moreover, TD is a persistent condition that is typically irreversible and difficult to treat. The standard of care includes the careful withdrawal of the antipsychotic medication, if feasible [7]. Alternatively, if the patient is taking a first-generation antipsychotic, they may be switched to a second-generation antipsychotic with lower affinity to D2 receptors [7]. More recently, VMAT2 inhibitors such as deutetrabenazine and valbenazine have been approved for the treatment of TD [7]. In contrast to TD, pseudo-tardive dyskinesia and WED have a transient impact on movement and resolve without interventions that would normally be used to treat TD. WED is generally limited to four to eight weeks and occurs in individuals who did not previously display dyskinetic symptoms during antipsychotic treatment [2]. Presently, there is a lack of consensus with respect to the diagnosis and management of WED [3]. Therefore, it is critical to remain mindful of the potential for withdrawal-emergent symptoms. Though self-limiting in most cases, the recommendation is that the offending antipsychotic be reinstituted and tapered gradually [2].

The patient in this case likely experienced a heightened effect of postsynaptic dopamine receptor hypersensitivity leading to his symptoms. This may be attributed to two simultaneous processes, the first process being the flooding of upregulated D2 receptors; in other words, increased sensitivity to endogenous dopamine. The second process being the increased pro-dopaminergic effect of the titration of methylphenidate as he reached his maximum dose on the same day as symptom onset. Methylphenidate exerts its effects at the level of the synaptic cleft by inhibiting the reuptake of norepinephrine and dopamine, thereby increasing the levels of these neurotransmitters. While the patient did have exposure to antipsychotic medication (two-month duration), and subsequent rapid withdrawal, the combined effect of a stimulant augmented the receptor hypersensitivity effects that are normally produced by antipsychotics alone. This synergistic effect observed in the setting of probable D2 receptor upregulation coupled with increased pro-dopaminergic stimulant effects represents a plausible and unique mechanism for dyskinesia. Additionally, the patient’s symptomatology may be ascribed to withdrawal EPS, given the temporal relationship between the time of antipsychotic discontinuation and symptom onset.

Another consideration to be made is the concomitant presence of a possible serotonin syndrome-like picture. Analogous to the mechanism of D2 receptor hypersensitivity outlined above, unopposed activation of 5HT receptors may have resulted from the rapid discontinuation of ziprasidone. Mechanistically, the long-term antagonism of 5HT receptors would have induced a compensatory upregulation of receptors. In addition to the reuptake inhibition activity of dopamine and norepinephrine, methylphenidate possesses agonist activity at the serotonin type 1A receptor [8]. This augmented serotonergic activity may have possibly produced a clinical scenario resembling that of serotonin syndrome. Clinically, this consideration was ruled out as our patient did not exhibit signs of autonomic instability nor did he present with altered mental status.

Management of this hypersensitivity dyskinesia would entail discontinuation of the antipsychotic and/or stimulant medication, both of which were discontinued in our patient but may have caused acute-onset symptom presentation due to rapid withdrawal and concurrent unopposed actions. While the patient was later initiated on bupropion, the literature has noted that the reuptake inhibition produced by bupropion is weaker than that produced by methylphenidate, in turn producing a weaker dopaminergic effect [9], which may have accounted for the remission of withdrawal symptoms as well as helped with regulating to a baseline state. The outcome of our patient’s management indicates that, in general, patients on concomitant antipsychotics and stimulant treatment regimens would benefit from a compensatory strategy that follows a slower taper of neuroleptics, as well as possible consideration of a cross-titration of a non-stimulant such as a norepinephrine-dopamine reuptake inhibitor or a non-dopaminergic agent such as atomoxetine or alpha-2 agonists while simultaneously discontinuing stimulants.

## Conclusions

WED is a transient movement disorder resulting from discontinuation or reduction in dosage of an antipsychotic medication. Contrary to TD, the existing literature on WED and other tardive syndromes is sparse. This report outlined a case of reversible oral dyskinesia in a 13-year-old male in the context of long-term neuroleptic use and its rapid withdrawal, coupled with stimulant titration (ziprasidone and methylphenidate). Using elements of the postsynaptic dopamine receptor hypersensitivity hypothesis, a potential pseudo-tardive conceptualization that aligns itself with withdrawal-emergent concepts should become a significant clinical consideration in management while treating the pediatric population given the high prevalence of comorbid diagnoses of DMDD and ADHD. From a clinical standpoint, this case may help better inform future treatment and management of these conditions and avert avoidable adverse side effects that have the potential to become irrevocable.
